# The Evolution of HIV-1 Diversity in Rural Cameroon and its Implications in Vaccine Design and Trials

**DOI:** 10.3390/v2020639

**Published:** 2010-02-12

**Authors:** Rebecca Powell, Denis Barengolts, Luzia Mayr, Phillipe Nyambi

**Affiliations:** 1 Department of Microbiology, New York University School of Medicine, New York, NY, USA; E-Mail: rebecca.powell@nyumc.org; 2 Department of Pathology, New York University School of Medicine, New York, NY, USA; 3 Veterans Affairs New York Harbor Healthcare Systems, New York, NY, USA

**Keywords:** HIV-1 Diversity, Rural Cameroon, phylogenetics

## Abstract

West-Central Africa is an epicenter of the HIV pandemic; endemic to Cameroon are HIV-1 viruses belonging to all (sub)subtypes and numerous Circulating Recombinant Forms (CRFs). The rural villages of Cameroon harbor many strains of HIV-1, though these areas are not as well monitored as the urban centers. In the present study, 82 specimens obtained in 2000 and 2001 from subjects living in the rural villages of the South and West Regions of Cameroon were subtyped in *gag*, *pol*, and *env* and compared to 90 specimens obtained in 2006–2008 in the same regions, in order to analyze HIV-1 evolution in these rural areas. It was found that in the South Region, the proportion of unique recombinant forms (URFs) remained constant (∼40%), while the amount of URFs containing fragments of a CRF increased by 25%. (Sub)subtypes A1, F2, H, and K, and CRF09_cpx, identified in 2000 and 2001, were replaced by CRFs 01_AE, 13_cpx, 14_BG, and 18_cpx in 2006–2008. In the West Region, (sub)subtypes A2, C, G, and H, and CRFs 01_AE and 09_cpx, identified in 2000–2001, were replaced by sub-subtype A1 and CRFs 25_cpx and 37_cpx in 2006–2008. The proportion of URFs in the West Region dropped significantly over the time period by 43%. In both Regions, the proportion of CRF02_AG increased at all loci. These findings demonstrate that the evolution of HIV-1 is distinct for each endemic region, and suggests that the proportion of URFs containing CRF fragments is increasing as the genetic identity of the virus continues to shift dramatically. This highlights the concern that subtype-specific vaccines may not be relevant in Cameroon, and that the distribution of viral diversity in these regions of Cameroon must be carefully monitored.

## 1. Introduction

As an epicenter of the HIV pandemic, the West-Central African country of Cameroon is host to one of the broadest genetic arrays of HIV viruses [1–8]. In addition to HIV-2 and HIV-1 group N and O viruses, all the group M subtypes have been found in Cameroon, and many Circulating Recombinant Forms (CRFs) of HIV-1 were first identified there [9–12]. CRFs arise from the transmission of Unique Recombinant Forms (URFs), which are the result of recombination brought about by dual HIV-1 infection – the concomitant or sequential infection by at least two distinct HIV-1 strains. Complicating the genetic landscape in Cameroon and elsewhere is the phenomenon of Second-Generation Recombinants (SGRs): URFs that contain fragments of one or more CRFs (such as CRF11_cpx, CRF22_01A1, and CRF36_cpx), as opposed to the initially-identified CRFs, which are generally mosaics of pure subtypes (such as CRFs 01_AE through 10_CD) [13]. Recently, we have found the frequency of dual infection in the urban center of Yaoundé to be 16% (∼11% incidence per year) in samples obtained between 2001 and 2004. This finding confirmed earlier data that had commonly identified URFs as the HIV-1 strains circulating among the HIV-positive population in the urban cores as well as certain rural areas [3,6,14,15]. This rate of dual infection coupled with the extremely diverse nature of the pandemic in Cameroon is cause for concern, as the design of relevant, effective vaccines may be severely compromised by such a genetic milieu.

A substantial body of data on HIV diversity in Cameroon has come from studies of the urban centers [6,14,16,17]. Phylogenetic study of the *gag* and *env* loci amplified from specimens obtained from subjects living in the cities of Yaoundé and Douala in 2002 by Machuca *et al*., found 60% of samples to be CRF02_AG, and 26% to be URFs; as well, 12 subtypes and CRFs were identified [6]. Recently, a comparative study of over 500 specimens obtained from calendar year 1996 to 2004 from urban, HIV-positive blood donors in Yaoundé and Douala found there to be no significant changes in the proportions of all but one subtype (increase in subtype D) over this time period, and that the strain compositions of the URFs did not shift significantly except in the case of a decrease in subtype G fragments [14].

Though rural settings comprise the majority of the country’s population, and in certain rural areas HIV prevalence has been found to be more than double the national rate, little data on HIV diversity in these regions exists, particularly data obtained in the last decade [1,18]. Most recently, a survey of rural villages of the East Region was conducted on samples obtained in 2000, which found CRF02_AG to be predominant among the *gag, pol,* and *env* gene fragments studied, and half of the specimens studied to be URFs, nearly all of which were SGRs containing CRF02_AG fragments [15]. Intriguingly, this survey led to the discovery of two novel CRFs in Cameroon, CRFs 36_cpx and 37_cpx, demonstrating the unique importance of proper monitoring of HIV diversity in these rural areas [10,11]. Similarly, another study conducted using a limited number of samples from villages in the East, Center, Southwest, and South Regions obtained in 2000 found CRF02_AG and subtype A viruses to be dominant, with numerous other subtypes co-circulating in these rural areas [2]. Further study of samples obtained in 2000–2001 from rural areas of the East, Center, South, Southwest, and West Regions also identified extremely broad subtype diversity, and in particular, revealed that URFs were exceedingly common (63% of samples) in villages of the West Region [3].

To date, no study of the evolution of HIV subtype diversity in the rural countryside has been reported. As such there is little information on the genetic landscape of current HIV infections in such regions with record high HIV-1 viral diversity – a concerning fact with regards to appropriate vaccine development. In the present study, the HIV-1 genetic subtypes infecting individuals in calendar year 2000–2001 in the rural villages of the South and West Regions of Cameroon were compared to those in calendar year 2006–2008 in the same geographic areas, in order to analyze HIV-1 evolution over this time period.

## 2. Results and Discussion

**Figure 1.**
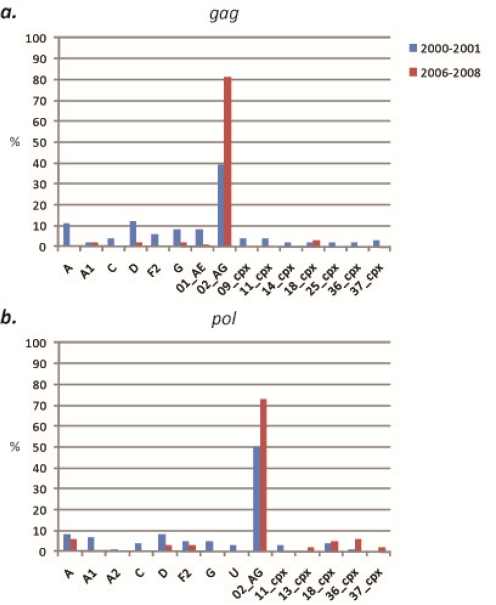

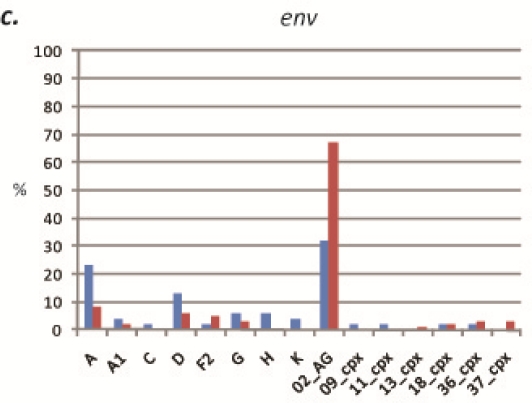
Percentages of each (sub)subtype and CRF at each locus, 2000–2001 and 2006–2008. **(a)** *gag*, 2000–2001 *vs.* 2006–2008. **(b)** *pol*, 2000–2001 *vs.* 2006–2008. **(c)** *env,* 2000–2001 *vs.* 2006–2008. Classifications are based on phylogenetic analysis using neighbor-joining and the Kimura 2-parameter method with bootstrapping (1000 replicates).

A total of 82 specimens obtained in the South and West regions were analyzed in calendar year 2000–2001 and identified nine (sub)subtypes (A, A1, A2, C, D, F2, G, H, and K), and 6 CRFs (01_AE, 02_AG, 09_cpx, 11_cpx, 18_cpx, and 36_cpx) as either pure strains (67% of all specimens, 55/82) or as URFs (52% of specimens evaluated at more than one locus, 27/52), based on phylogenetic analysis of *gag*, *pol*, and *env* fragments (Figure 1).

### 2.1. HIV-1 genetic diversity by geographic region identified in calendar year 2000–2001

#### 2.1.1. South Region

**Figure 2.**
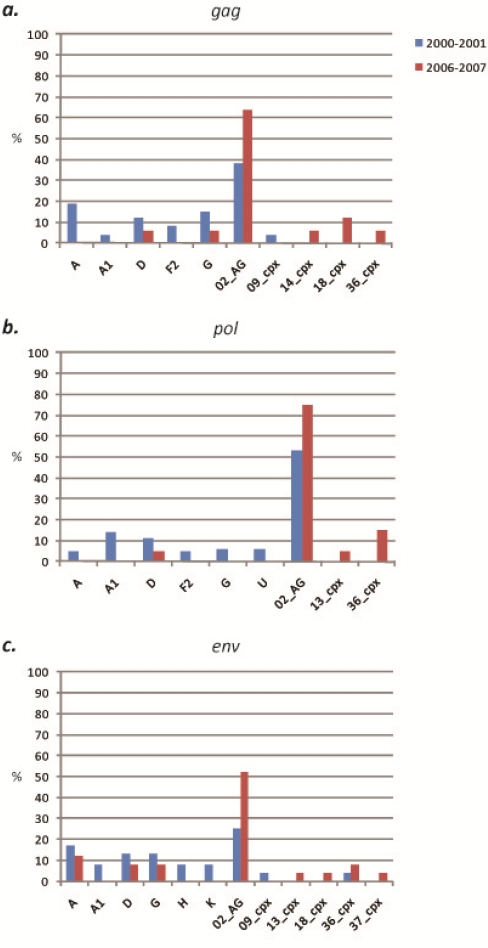
Percentages of each (sub)subtype and CRF at each locus, South region. **(a)** *gag*, 2000–2001 *vs.* 2006–2007. **(b)** *pol*, 2000–2001 *vs.* 2006–2007. **(c)** *env,* 2000–2001 *vs.* 2006–2007.

**Figure 3.**
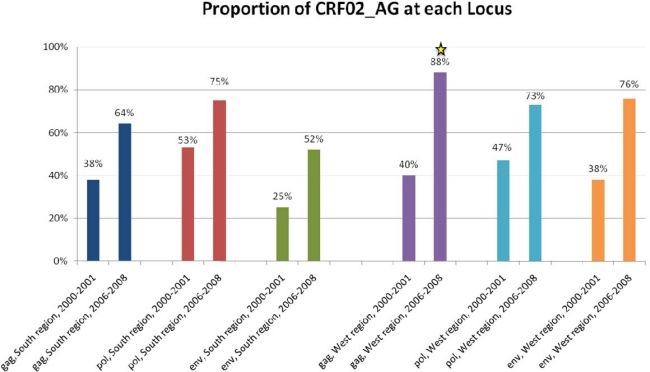
Proportion of CRF02_AG at each locus, South and West regions. Statistically significant increase (p < 0.05) is denoted by an asterisk.

Forty specimens acquired in 2000 and 2001 from subjects living in the rural villages of the South region were evaluated. Sequences were successfully obtained for 65% (26/40) of samples in *gag*, 90% (36/40) of samples in *pol,* and 60% (24/40) of samples in *env*. Among the 3 gene fragments analyzed, 10 (sub)subtypes and CRFs were identified, including (sub)subtypes A, A1, D, F2, G, H, K and CRFs 02_AG, 09_cpx, and 36_cpx (Figure 2). CRF02_AG was consistently found to be the dominant strain in each gene, comprising 38% (10/26), 53% (19/36), and 25% (6/24) of specimens at the *gag*, *pol*, and *env* loci, respectively (Figure 3). Among the non-02_AG *gag* sequences, 19% (5/26) clustered in the subtype A radiation but not closely with the known A sub-subtypes, 15% (4/26) clustered with subtype G, 11% (3/26) clustered with subtype D, 8% (n = 2) clustered with sub-subtype F2, and 4% of sequences (1/26) each clustered with sub-subtype A1 and CRF09_cpx. Of the non-02_AG *pol* sequences, 14% (5/36) clustered with sub-subtype A1, 11% (4/36) clustered with subtype D, and 5% (2/36) of sequences each clustered with (sub)subtype A, F2, and G. Two specimens were found to be unclassifiable in *pol*. In *env,* the non-02_AG sequences were classified as follows: 17% (4/24) clustered with subtype A, 13% of sequences (3/24) each clustered with subtypes D and G, 8% of sequences (2/24) each clustered with (sub)subtypes A1, H, and K, and 4% of sequences (1/24) each clustered with CRF09_cpx and CRF36_cpx (Supplementary Table 1). Every sequence was analyzed individually and clustering of a test sequence with a subtype reference with a bootstrap value of more than 60% was used for subtyping.

**Figure 4.**
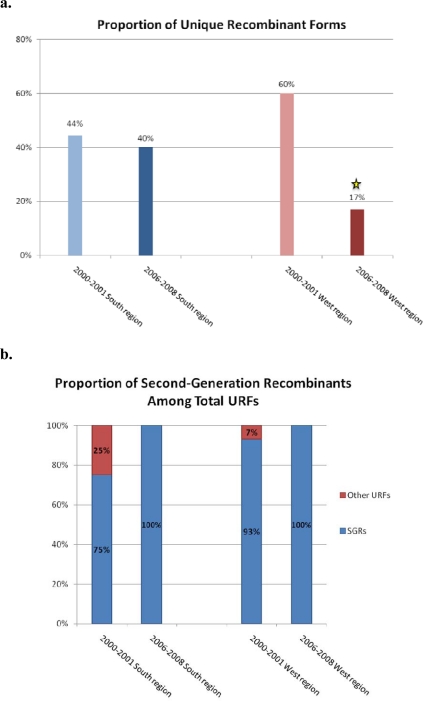
Proportion of Unique Recombinant Forms (URFs) and Second-Generation Recombinants (SGRs), South and West regions. **(a)** Percentages of URFs among total specimens. **(b)** Percentages of SGRs among total URFs. Statistically significant decrease (p < 0.05) is denoted by an asterisk.

Sixty-eight percent of specimens were analyzed at more than one region; therefore these samples were further evaluated for concordant or discordant combinations of subtypes, which provided an estimate of the proportion of unique recombinant strains (URFs). Of the 27 samples evaluated, 44% (n = 12) were shown to be URFs, 75% (9/12) of which included one or more fragments of a previously-identified CRF (*i.e.*, an SGR). Eight of the nine SGRs contained fragments of CRF02_AG and the remaining one SGR contained a fragment of CRF36_cpx (Figure 4).

#### 2.1.2. West Province

**Figure 5.**
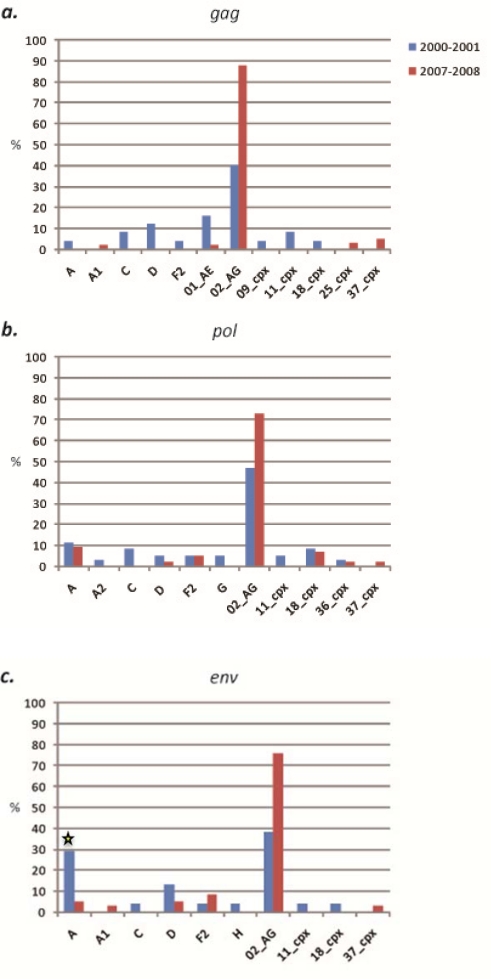
Percentages of each (sub)subtype and CRF at each locus, West region. **(a)** *gag*, 2000–2001 *vs.* 2007–2008. **(b)** *pol*, 2000–2001 *vs.* 2007–2008. **(c)** *env,* 2000–2001 *vs.* 2007–2008. Statistically significant decrease (p < 0.05) is denoted by an asterisk.

Forty-two specimens acquired in 2000 and 2001 in the rural villages of the West Region were evaluated. Sequences were obtained for 59% (25/42) of samples in *gag*, 90% (38/42) of samples in *pol,* and 57% (24/42) of samples in *env*. Among the three loci, 13 (sub)subtypes and CRFs were identified, including (sub)subtypes A, A2, C, D, F2, G, and H, and CRFs 01_AE, 02_AG, 09_cpx, 11_cpx, 18_cpx, and 36_cpx (Figure 5). CRF02_AG was the dominant strain in each gene, comprising 40% (10/25), 47% (18/38), and 38% (9/24) of specimens at the *gag*, *pol*, and *env* loci, respectively (Figure 3). Of the remaining sequences in *gag*, 16% (4/25) clustered with CRF01_AE, 12% (n = 25) clustered with subtype D, 8% (n = 25) each clustered with subtype C and CRF11_cpx, and 4% of sequences (n = 25) each clustered with subtypes A, F2, and CRFs 09_cpx and 18_cpx. In *pol*, 11% (4/38) of the non-02_AG specimens clustered in the subtype A radiation, 8% of sequences (3/38) each clustered with subtype C and CRF18_cpx, 5% (2/38) each clustered with subtypes D, F2, and G and CRF11_cpx, and 3% (1/38) each clustered with sub-subtype A2, and CRF36_cpx. In *env*, the remaining sequences were classified as: 29% (7/24) subtype A, 13% (3/24) subtype D, and 4% of sequences (1/24) each clustered with subtypes C, F2, H, and CRFs 11_cpx and 18_cpx (Supplementary Table 1). Every sequence was analyzed individually and clustering of a test sequence with a subtype reference with a bootstrap value of more than 60% was used for subtyping.

Sixty percent (25/40) of specimens were analyzed at more than one region; therefore these samples were further evaluated for concordant or discordant combinations of subtypes. It was found that 60% (15/25) were URFs, 93% (14/15) of which were SGRs. The SGRs were typically comprised of fragments of CRF02_AG (n = 10), while a minority included CRF01 (n = 4), CRF11 (n = 1), and CRF36 (n = 1) fragments (Figure 4).

### 2.2. HIV-1 genetic diversity by geographic region identified in calendar year 2006–2008

A total of 90 specimens obtained in the South and West regions were analyzed in calendar year 2006–2008 and identified five (sub)subtypes (A, A1, D, F2, and G) and nine CRFs (01_AE, 02_AG, 11_cpx, 13_cpx, 14_BG, 18_cpx, 25_cpx, and 36_cpx, and 37_cpx), as either pure strains (80% of all specimens, 72/90) or as URFs (26% of specimens evaluated at more than one locus, 18/70), based on phylogenetic analysis of *gag*, *pol*, and *env* fragments (Figure 1).

#### 2.2.1. South Region

Twenty-eight specimens acquired in 2006 and 2007 in the rural villages of the South Region from subjects different from those of the 2000–2001 analysis were evaluated. Sequences were obtained for 61% (17/28) of samples in *gag*, 71% (20/28) of samples in *pol,* and 89% (25/28) of samples in *env*. Nine (sub)subtypes and CRFs were identified, including subtypes A, D, and G, and CRFs 01_AE, 02_AG, 13_cpx, 14_BG, 18_cpx, and 36_cpx (Figure 2). CRF02_AG was again found to be the dominant strain in each gene, comprising 65% (11/17), 75% (15/20), and 52% (13/25) of specimens at the *gag*, *pol*, and *env* loci, respectively. These increases in CRF02_AG compared to the 2000–2001 specimens were not found to be statistically significant (two-way chi-square test, 95% confidence intervals) (Figure 3). In *gag,* the next most common strain identified was CRF18_cpx (12%, 2/17), followed by subtypes D and G, and CRFs 13_cpx, 14_BG, and 36_cpx, each comprising 6% (1/17) of the sequences. In *pol*, 15% (3/20) of the remaining sequences clustered with CRF36_cpx, 7% (2/20) clustered with CRF18_cpx, and 5% of sequences (1/20) each clustered with subtype D and CRF13_cpx. Of the non-02_AG sequences in *env*, 12% (3/25) of sequences clustered with subtype A, 8% of sequences (2/25) each clustered with subtypes D and G and CRF36_cpx, and 4% of sequences (1/25) each clustered with CRFs 13_cpx, 18_cpx, and 37_cpx (Supplementary Table 2). Every sequence was analyzed individually and clustering of a test sequence with a subtype reference with a bootstrap value of more than 60% was used for subtyping.

Eighty-nine percent of specimens (n = 25) were analyzed at one or more region; therefore these samples were further evaluated for concordant or discordant combinations of subtypes. It was found that 40% (n = 10) were URFs, 100% of which were SGRs. These SGRs were comprised of fragments of CRF02_AG (n = 9), CRF36 (n = 4), CRF18 (n = 2), CRF13 (n = 1), and CRF14 (n = 1) (Figure 4).

In the South Region over this 7–8 year period, the proportion of unique recombinant forms (URFs) remained constant, while the amount of SGRs increased by 25%. (Sub)subtypes A1, F2, H, and K, and CRF09_cpx, identified in 2000–2001, were replaced by CRFs 01_AE, 13_cpx, 14_BG, and 18_cpx in 2006–2008.

#### 2.2.2. West Region

Sixty-two specimens acquired in 2007 and 2008 in the rural villages of the West region from subjects different from the 2000–2001 analysis were evaluated. Sequences were obtained for 68% (41/62) of samples in *gag*, 73% (44/62) of samples in *pol,* and 63% (38/62) of samples in *env*. Ten (sub)subtypes and CRFs were identified, including (sub)subtypes A, A1, D, and F2, and CRFs 02_AG, 11_cpx, 18_cpx, 25_cpx, 36_cpx, and 37_cpx. (Figure 5). CRF02_AG was found to be the dominant strain in each gene, comprising 88% (36/41), 73% (32/44), and 76% (29/38) of specimens at the *gag*, *pol*, and *env* loci, respectively (Figure 3). The increase in CRF02_AG at *gag* compared to the 2000–2001 specimens was statistically significant (p = 0.03). The non-02_AG *gag* sequences were classified as 5% (2/41) CRF37_cpx, and 2% each (1/41) of sub-subtype A1, and CRFs 01_AE and 25_cpx. The decreases in both subtype D and CRF01_AE in *gag* compared to the 2000–2001 specimens were statistically significant (p = 0.02 and p = 0.04, respectively). In *pol*, 9% (4/44) of the non-02_AG sequences clustered with subtype A, 7% (3/44) clustered with CRF18_cpx, 5% (2/44) clustered with sub-subtype F2, and 2% (1/44) each clustered with subtype D, and CRFs 36_cpx and 37_cpx. In *env*, 8% (3/38) of the remaining sequences clustered with sub-subtype F2, 5% of sequences (2/38) each clustered with subtypes A and D, and 3% of sequences (1/38) each clustered with sub-subtype A1 and CRF37_cpx (Supplementary Table 2). The decrease in subtype A as compared to the 2000–2001 specimens was statistically significant (p = 0.01).

Seventy-three percent (45/62) of specimens were analyzed at more than one region. It was found that 17% (8/45) were URFs, all of which were SGRs. This decline in the proportion of URFs in the West region over the study period was found to be highly significant (p = 0.003). The SGRs were comprised of fragments of CRF02_AG (n = 6), CRF18 (n = 3), CRF36 (n = 1), CRF01 (n = 1), and CRF25 (n = 1) (Figure 4).

In the West Region over the 7–8 year period, (sub)subtypes A2, C, G, and H, and CRFs 01_AE and 09_cpx, identified in 2000–2001, were replaced by sub-subtype A1 and CRFs 25_cpx and 37_cpx in 2006–2008. The proportion of URFs in the West Region dropped significantly over the time period by 43%. Significant decreases were found in subtypes A (at *env*) and D and CRF01_AE (at *gag*), and a significant increase in CRF02_AG (at *gag*) was identified.

### 2.3. Discussion

It is clear from the analysis of both regions of Cameroon that the genetic character of HIV-1 has continued to shift over the time period studied, and that this evolution is unique for each geographic area. While in the South Region, the proportion of URFs remained relatively constant between 2000–2001 (44%) and 2006–2008 (40%), in the West Region there was a statistically significant decrease of URFs from 60% in time period 2000–2001 to 17% in 2007–2008. Both regions showed increases in the proportion of SGRs over the time period: 25% more SGRs in the South and 7% more SGRs in the West, increases that left both regions with SGRs comprising 100% of the URFs identified. Although most of these changes in URF and SGR proportions were not significant, they may be an indication of growing trends to be closely monitored in future surveys. As only three genomic fragments were studied herein, it should be noted that these are only the minimum proportions of URFs and SGRs possible. Full-length sequencing might have revealed additional recombination in the specimens considered here to be pure (sub)subtypes or CRFs. Therefore, the true frequency of URF’s is likely to be even higher than identified in this study. Samples of interest, such as those with discordant subtype designations in different gene segments, will be further analyzed in order to reveal the true frequency of URF’s.

Recombinant forms of HIV-1 arise as a result of dual infection – the concomitant or sequential infection by two or more strains of HIV-1. The stability of URFs in the South region and the significant decrease of URFs in the West region suggest that the rate of dual infection may have remained constant or decreased over the 7–8 years time period studied. It is also possible that the URFs generated by these infections were poorly transmissible and/or comparably unfit among the co-circulating HIV-1 in these areas. We have found the frequency of dual infection among samples obtained between calendar years 2001–2004 in the urban center of Cameroon to be 16%; studies of dual infection as well as the biologic properties of viruses in these rural villages over the time period studied herein are warranted to better understand the driving factors in the genetic evolution of these viruses.

Although the number of different (sub)subtypes and CRFs identified remained fairly constant in both regions of Cameroon over the time period, it was evident that this did not correspond to static diversity. On the contrary, there were obvious shifts in genetic identity in both regions. In the South, (sub)subtypes A1, F2, H, and K, and CRF09_cpx, identified in 2000–2001, were replaced by CRFs 01_AE, 13_cpx, 14_BG, and 18_cpx in the 2006–2007 specimens. In the West, (sub)subtypes A2, C, G, and H, and CRFs 01_AE and 09_cpx, identified in 2000–2001, were replaced by sub-subtype A1 and CRFs 25_cpx and 37_cpx in 2007–2008. Although most of these shifts in subtype identity and proportion were not significant statistically, they do signify that larger studies of rural villages are necessary to properly characterize these endemic areas. Furthermore, in both regions, the proportion of CRF02_AG had increased at all loci, though only the increase in the West region at *gag* was significant (48%).

These increases of CRF02_AG suggest that HIV-1 is becoming more genetically homogenous in both the South and West regions. This may appear as particularly encouraging with respect to the *env* locus, as homogeneity in *env* would certainly aid in the design and efficacy of novel vaccines. However, considering the abundantly diverse HIV-1 strains still found to be co-circulating in both regions, and the general shift in HIV-1 diversity, especially with the emergence of several SGRs that show high levels of recombination with CRF02_AG variants within the relatively short time period, it is clear that the dominance of CRF02_AG may reduce over time.

The recent study by Brennan *et al*., in which over 500 specimens obtained from 1996–2004 from urban, HIV-positive blood donors in Yaoundé and Douala were analyzed, found there to be no significant changes in the proportions of all but one subtype (D) over the 8-year period, and that the strain compositions of the URFs identified changed significantly only for the proportion containing subtype G [14]. Despite the small sample size analyzed herein relative to the Brennan *et al*. study, our survey of these rural areas found significant decreases in subtypes A (at *env*) and D and CRF01_AE (at *gag*) in the West region and a significant increase in CRF02_AG (at *gag*), in addition to the overall significant decrease in URFs, suggesting HIV-1 evolution in these rural areas to be more marked than in the urban centers. These results imply that larger sample sizes in future studies of rural Cameroon may demonstrate an even more dynamic HIV population, and highlights the concern that subtype-specific vaccines may not be relevant in these areas of Cameroon, and that the distribution of viral diversity in these regions must be carefully monitored.

## 3. Experimental Section

### 3.1. Study subjects

Blood samples from the general population from 82 HIV-positive, asymptomatic, chronically-infected subjects were collected in calendar years 2000 and 2001 in rural villages of the South and West Regions of Cameroon. The 40 samples collected in the South region were from 15 men and 25 women with ages ranging from 21 to 56 years (mean, 37 years). Thirty-four of these specimens were included in a previous study of the South region [2,3,19]. Forty-two specimens (19 men, 22 women, one unavailable) were obtained in the West region, 28 of which were subjects of previous study of the West region [2,3,19]. These study subjects were between 17 and 70 years of age (mean, 36 years). In calendar years 2006–2008, 95 HIV-positive blood samples were collected from additional subjects living in the same regions. The 30 specimens obtained in the South region were from seven men and 21 women (information on two subjects was unavailable) who were between 21 and 62 years of age (mean, 34 years), while the 65 samples from the West region were from 12 men and 24 women (information on 22 subjects was unavailable) who were between 18 and 64 years of age (mean, 35 years). The samples from the South province were obtained from the following villages: Endengue, Minko’o, Yen, Akom, Nkono-Gbas, Bitche, Andoung, Mekas, Olembe, Melombo, Ndtoua, Ebodje, Idondefang, Mabiogo, Bidjouka, Mveng, Djoum and Efoulam. The samples from the West province were collected in the following villages: Biassamba, Makoutam, Makoutmbou, Makpa, Mahoua, Matambou, Nabouobou, Manje Magni, Marapdoum, Tchouadeng, Junitsa, Mapoure and Malantouen. All study subjects were antiretroviral drug naïve. Summarized study subject data is available in Supplementary Tables 1 and 2.

### 3.2. RNA Extraction, Reverse Transcriptase-Polymerase Chain Reaction and Sequencing

Plasma was obtained by Ficoll-Hypaque gradient centrifugation of whole blood. Viral RNA was extracted from plasma using the QIAamp Viral RNA Mini kit (Qiagen Inc, Valencia, CA). 2 μl of RNA was used for one-tube reverse transcriptase polymerase chain reaction (RT-PCR) using the Superscript One-Step RT-PCR for Long Templates kit (Invitrogen, Carlsbad, CA) to amplify a fragment of *gag* (HXB2 positions 1577–2040, 463 bp), part of the *pol* gene encoding the protease (PR) enzyme (HXB2 positions 2241–2577, 336 bp), as well as the C2-V5 region of e*nv* (HXB2 positions 7001–7647, 646 bp). 2 μl of first-round product was then used in a nested PCR using the Platinum PCR SuperMix High Fidelity system (Invitrogen, Carlsbad, CA). The gag fragment was amplified using the outer primers H1G777 and H1P202 and the inner primers H1gag1584 and G17 [20]. The PR region of *pol* was amplified using the outer primers NYUPOL6, NYUPOL7 and NYUPOL8 and the inner primers NYUPOL9 and NYUPOL10 [19]. All the primers have previously been described. The amplification conditions for the gag and pol fragments for the one-tube RT-PCR was one cycle of 50 °C for 30 minutes and 94 °C for 2 minutes, followed by 40 cycles of 94 °C for 15 seconds, 50 °C for 30 seconds and 68 °C for 1 minute, ending with a single extension cycle at 72 °C for 7 minutes. For the second-round PCR, the above protocol was used for 35 cycles, beginning with the 94 °C denaturation step. To amplify the C2-V5 region of *env* the previously described outer primers ED5 and ED12 and the inner primers ES7 and ES8 were used [21]. The amplification conditions for the env fragment for the one-tube RT-PCR was one cycle of 50 °C for 30 minutes, followed by 3 cycles of 94 °C, 55 °C, and 72 °C each for 1 minute. This was followed by 32 cycles of 94 °C for 15 seconds, 55 °C for 45 seconds and 72 °C for 1 minute, ending with a single extension cycle at 72 °C for 7 minutes. For the second-round PCR, the above protocol was repeated without the RT step. Polymerase chain reaction products were directly sequenced at the 5′and 3′ ends using the respective outer primers.

### 3.3. Phylogenetic Analysis

The sequences were automatically aligned with identical regions of reference sequences of all known HIV-1 group M (sub-)subtypes and CRFs from the Los Alamos HIV database using CLUSTAL X, and manually cropped using Seaview [13,22,23]. Phylogenetic analyses were conducted using MEGA version 4 software package [24]. Pairwise evolutionary distances were estimated using Kimura’s two-parameter method, and phylogenetic trees were constructed by neighbor-joining [25,26]. Clustering of sequences with a bootstrap value of more than 60% was used for subtyping. All sequences were included in a single phylogenetic tree for each locus in order to identify and omit highly related sequences and avoid study subject resampling. No such sequences were found.

Differences in the proportions of each (sub)subtype or CRF at each locus, and of URFs and SGRs at each study period for each geographic region were evaluated using a Two-way chi-square test (95% confidence interval).

### 3.4. Accession numbers

The sequences generated for this study are available from GenBank with the accession numbers GU047883–GU048094.

## 4. Conclusion

In this report, we observed that CRF02_AG in the rural South and West regions of Cameroon remained dominant over a 7–8 period, that various co-circulating HIV-1 subtypes and CRFs identified in 2000–2001 were replaced by others by 2006–2008, and that the proportions of SGRs in both regions increased. Importantly, these SGRs were mainly variants of CRF02_AG that had recombined with other HIV-1 subtypes. These findings demonstrate that even in this short time period, the genetic identity of the virus continues to shift dramatically, suggesting that the viral diversity in these rural regions may become more complex, that any subtype specific vaccine may not be relevant in such regions of Cameroon, and that the distribution and evolution of viral diversity in these regions of Cameroon must be monitored. Therefore, a vaccine that would be effective in these regions of the world must target conserved antigenic regions shared by diverse HIV-1 strains.

## Supplementary Materials
