# Supplementary material for: The Evolution of HIV-1 Diversity in Rural Cameroon and its Implications in Vaccine Design and Trials
Source: Viruses. 2010 Feb 12;2(2):639–54. doi: 10.3390/v2020639 (PMC2975583; doi:10.3390/v2020639)
Supplement: Supplementary file 1 [file viruses-02-00639-s001.pdf]

Supplementary Tables for article "The Evolution of HIV-1 Diversity in Rural Cameroon and its Implications in Vaccine Design and Trials", published 11 February 2010.

Supplementary Table 1. Study Subject Data, 2000–2001.

				**Subtype**		
**Sample ID**	**Age**	**Sex**	**Region**	***Gag***	***Pol***	***Env***
00CMNYU1423	38	F	South	A	A	A
00CMNYU1457	33	F	South	D	D	D
00CMNYU1500	24	F	South	A	A	A
00CMNYU1545	34	F	South	D	D	D
00CMNYU1557	28	F	South	-	02_AG	G
00CMNYU1573	41	F	South	A	-	02_AG
00CMNYU1591	44	M	South	02_AG	02_AG	02_AG
00CMNYU1607	56	F	South	02_AG	02_AG	02_AG
00CMNYU1678	48	M	South	G	G	G
00CMNYU1678	48	M	South	G	G	G
00CMNYU1705	41	M	South	D	D	D
00CMNYU1725	32	F	South	02_AG	02_AG	02_AG
00CMNYU1725	32	F	South	-	02_AG	-
00CMNYU1748	48	M	South	-	02_AG	-
00CMNYU1749	30	M	South	02_AG	-	36_cpx
00CMNYU1818	32	F	South	A1	A1	A1
00CMNYU1819	35	F	South	02_AG	-	K
00CMNYU1840	53	F	South	-	02_AG	-
00CMNYU1911	33	F	South	-	D	-
00CMNYU1978	33	M	South	G	A1	H
00CMNYU1989	25	F	South	G	U	H
00CMNYU1999	23	F	South	F2	F2	-
00CMNYU2031	34	M	South	-	A1	-
00CMNYU2113	27	M	South	09_cpx	-	09_cpx
00CMNYU2124	47	F	South	02_AG	02_AG	-
00CMNYU2140	50	F	South	F2	02_AG	-
00CMNYU2192	30	F	South	02_AG	02_AG	K
00CMNYU2201	55	F	South	-	02_AG	-
00CMNYU2224	27	F	South	-	02_AG	-
00CMNYU2276	46	M	South	-	02_AG	-
00CMNYU2362	40	M	South	02_AG	02_AG	A1
00CMNYU2389	37	M	South	-	F2	-
00CMNYU2395	21	F	South	02_AG	02_AG	02_AG
00CMNYU2445	35	M	South	-	A1	-
00CMNYU2473	44	F	South	-	02_AG	-
00CMNYU2636	32	M	South	A	U	A
00CMNYU2663	40	F	South	A	A1	02_AG
00CMNYU2845	23	F	South	02_AG	02_AG	A
00CMNYU2878	30	F	South	-	02_AG	-
00CMNYU2987	38	M	South	-	02_AG	-

Supplementary Table 1. *Cont.*

00CMNYU4877	17	F	West	D	D	D
00CMNYU5056	35	F	West	-	02_AG	-
01CMNYU5079	37	M	West	-	02_AG	-
01CMNYU5149	27	F	West	-	F2	-
01CMNYU5184	43	M	West	02_AG	A	02_AG
01CMNYU5203	70	M	West	02_AG	02_AG	02_AG
01CMNYU5218	70	M	West	C	C	C
01CMNYU5235	38	F	West	-	C	-
01CMNYU5274	28	F	West	-	G	-
01CMNYU5285	32	M	West	-	G	A
01CMNYU5308	51	M	West	11_cpx	11_cpx	A
01CMNYU5337	29	F	West	C	02_AG	02_AG
01CMNYU5346	34	M	West	11_cpx	11_cpx	11_cpx
01CMNYU5423	52	M	West	-	C	-
01CMNYU5466	19	F	West	02_AG	02_AG	02_AG
01CMNYU5467	42	M	West	-	02_AG	-
01CMNYU5487	58	M	West	01_AE	A	A
01CMNYU5497	20	F	West	-	02_AG	-
01CMNYU5515	36	F	West	-	02_AG	-
01CMNYU5581	29	M	West	-	02_AG	-
01CMNYU5630	21	F	West	-	02_AG	-
01CMNYU5685	45	M	West	-	A2	-
01CMNYU5795	41	M	West	-	02_AG	-
01CMNYU5821	38	M	West	A	02_AG	H
01CMNYU5854	26	F	West	-	02_AG	-
01CMNYU5855	28	M	West	02_AG	02_AG	A
01CMNYU5887	35	M	West	F2	F2	F2
01CMNYU6078	40	F	West	02_AG	02_AG	A
01CMNYU6283	24	F	West	01_AE	A	A
01CMNYU6294	22	F	West	18_cpx	18_cpx	18_cpx
01CMNYU6738	62	M	West	02_AG	18_cpx	02_AG
01CMNYU6741	24	M	West	02_AG	18_cpx	02_AG
01CMNYU6745	40	F	West	01_AE	A	-
01CMNYU6747	26	F	West	D	D	D
01CMNYU6773	40	F	West	02_AG	02_AG	02_AG
01CMNYU6783	41	M	West	D	-	D
01CMNYU6826	38	F	West	-	-	02_AG
01CMNYU6830	40	F	West	09_cpx	-	-
01CMNYU6853	42	F	West	02_AG	-	-
01CMNYU6869	32	F	West	01_AE	02_AG	-
01CMNYU6951	19	F	West	02_AG	36_cpx	02_AG
01CMNYU7100	NA	NA	West	-	02_AG	A

NA: Not available. A dash (-) indicates the sequence was not obtained.

Grey shading: SGR. Green shading: other URF.

Supplementary Table 2. Study Subject Data, 2006–2008.

					Subtype	
**Sample ID**	**Sex**	**Age**	**Region**	***Gag***	***Pol***	***Env***
06CMNYU015	F	23	South	36_cpx	02_AG	36_cpx
06CMNYU027	F	18	South	02_AG	02_AG	A
06CMNYU035	F	46	South	18_cpx	02_AG	02_AG
06CMNYU039	F	22	South	02_AG	02_AG	-
06CMNYU045	F	23	South	-	02_AG	02_AG
06CMNYU105	F	38	South	02_AG	02_AG	02_AG
06CMNYU117	F	47	South	02_AG	-	02_AG
06CMNYU137	M	57	South	18_cpx	02_AG	18_cpx
06CMNYU140	F	20	South	G	13_cpx	13_cpx
06CMNYU187	F	32	South	02_AG	02_AG	02_AG
06CMNYU195	F	24	South	02_AG	-	02_AG
06CMNYU204	M	27	South	02_AG	-	36_cpx
06CMNYU250	F	24	South	14_cpx	02_AG	G
07CMNYU496	F	29	South	-	36_cpx	-
07CMNYU497	F	28	South	02_AG	36_cpx	-
07CMNYU498	F	36	South	-	-	D
07CMNYU499	F	59	South	-	02_AG	A
07CMNYU500	M	34	South	02_AG	02_AG	02_AG
07CMNYU501	F	51	South	D	D	D
07CMNYU503	F	19	South	02_AG	36_cpx	A
07CMNYU506	F	49	South	02_AG	-	02_AG
07CMNYU507	M	62	South	-	-	37_cpx
07CMNYU510	M	32	South	-	02_AG	02_AG
07CMNYU512	F	NA	South	-	02_AG	02_AG
07CMNYU513	M	32	South	-	-	G
07CMNYU515	F	33	South	-	02_AG	02_AG
07CMNYU516	F	21	South	-	-	02_AG
07CMNYU518	M	33	South	-	02_AG	02_AG
07CMNYU370	F	19	West	02_AG	36_cpx	A1
07CMNYU378	M	47	West	02_AG	18_cpx	02_AG
07CMNYU381	F	64	West	-	02_AG	-
07CMNYU437	M	36	West	-	02_AG	02_AG
07CMNYU456	F	25	West	02_AG	02_AG	02_AG
07CMNYU471	M	42	West	37_cpx	37_cpx	37_cpx
07CMNYU472	F	23	West	37_cpx	-	-
07CMNYU473	F	18	West	A1	A	-
07CMNYU474	M	27	West	-	02_AG	02_AG
07CMNYU475	F	44	West	-	-	D
07CMNYU476	F	45	West	-	-	D
07CMNYU477	M	42	West	-	02_AG	02_AG
07CMNYU480	F	53	West	-	-	02_AG
07CMNYU481	F	18	West	-	02_AG	02_AG
07CMNYU482	F	23	West	-	02_AG	-
07CMNYU484	NA	NA	West	-	-	02_AG
07CMNYU487	NA	NA	West	-	18_cpx	02_AG

Supplementary Table 2. *Cont.*

07CMNYU488	NA	NA	West	02_AG	18_cpx	02_AG
07CMNYU489	NA	NA	West	02_AG	02_AG	-
07CMNYU490	NA	NA	West	02_AG	-	-
07CMNYU491	NA	NA	West	02_AG	02_AG	02_AG
07CMNYU492	NA	NA	West	02_AG	-	-
08CMNYU536	NA	NA	West	02_AG	-	-
08CMNYU593	M	42	West	02_AG	02_AG	02_AG
08CMNYU603	M	27	West	02_AG	-	02_AG
08CMNYU605	F	35	West	02_AG	A	02_AG
08CMNYU607	F	26	West	02_AG	-	02_AG
08CMNYU700	NA	NA	West	-	F2	F2
08CMNYU701	NA	NA	West	-	D	-
08CMNYU707	M	41	West	02_AG	02_AG	02_AG
08CMNYU725	F	23	West	02_AG	02_AG	-
08CMNYU813	F	27	West	02_AG	02_AG	-
08CMNYU871	F	46	West	02_AG	02_AG	-
08CMNYU927	F	32	West	02_AG	02_AG	02_AG
08CMNYU958	F	20	West	02_AG	-	-
08CMNYU962	F	38	West	02_AG	02_AG	02_AG
08CMNYU993	NA	NA	West	-	-	02_AG
08CMNYU995	NA	NA	West	02_AG	02_AG	-
08CMNYU997	M	45	West	02_AG	02_AG	A
08CMNYU998	M	19	West	02_AG	-	02_AG
08CMNYU999	NA	NA	West	02_AG	02_AG	-
08CMNYU1001	NA	NA	West	-	02_AG	-
08CMNYU1003	NA	NA	West	02_AG	02_AG	02_AG
08CMNYU1004	NA	NA	West	-	-	02_AG
08CMNYU1005	NA	NA	West	02_AG	02_AG	02_AG
08CMNYU1006	F	19	West	02_AG	02_AG	A
08CMNYU1007	F	25	West	02_AG	02_AG	02_AG
08CMNYU1008	M	27	West	-	F2	F2
08CMNYU1011	NA	NA	West	02_AG	02_AG	-
08CMNYU1012	NA	NA	West	02_AG	02_AG	-
08CMNYU1013	NA	NA	West	02_AG	02_AG	-
08CMNYU1079	M	88	West	02_AG	02_AG	-
08CMNYU1108	M	52	West	-	A	-
08CMNYU1112	F	38	West	02_AG	02_AG	-
08CMNYU1116	F	26	West	01_AE	A	-
08CMNYU1118	F	26	West	-	02_AG	-
08CMNYU1119	NA	NA	West	02_AG	02_AG	02_AG
08CMNYU1121	NA	NA	West	02_AG	-	02_AG
08CMNYU1122	F	38	West	25_cpx	-	F2
08CMNYU1125	F	60	West	-	-	02_AG
08CMNYU1149	F	22	West	02_AG	02_AG	02_AG
08CMNYU1150	F	32	West	-	-	02_AG

NA: Not available. A dash (-) indicates the sequence was not obtained.

Grey shading: SGR. Green shading: other URF.
